# Recent Advances in Biotechnologies for the Treatment of Environmental Pollutants Based on Reactive Sulfur Species

**DOI:** 10.3390/antiox12030767

**Published:** 2023-03-21

**Authors:** Kaili Fan, Wei Wang, Xijun Xu, Yuan Yuan, Nanqi Ren, Duu-Jong Lee, Chuan Chen

**Affiliations:** 1 State Key Laboratory of Urban Water Resource and Environment, School of Environment, Harbin Institute of Technology, Harbin 150090, China; 2 College of Biological Engineering, Beijing Polytechnic, Beijing 100176, China; 3 Department of Chemical Engineering, National Taiwan University, Taipei 106, Taiwan

**Keywords:** reactive sulfur species, microorganisms, environmental technology, wastewater treatment

## Abstract

The definition of reactive sulfur species (RSS) is inspired by the reactivity and variable chemical valence of sulfur. Sulfur is an essential element for life and is a part of global geochemical cycles. Wastewater treatment bioreactors can be divided into two major categories: sulfur reduction and sulfur oxidation. We review the origins of the definition of RSS and related biotechnological processes in environmental management. Sulfate reduction, sulfide oxidation, and sulfur-based redox reactions are key to driving the coupled global carbon, nitrogen, and sulfur co-cycles. This shows the coupling of the sulfur cycle with the carbon and nitrogen cycles and provides insights into the global material−chemical cycle. We also review the biological classification and RSS metabolic mechanisms of functional microorganisms involved in the biological processes, such as sulfate-reducing and sulfur-oxidizing bacteria. Developments in molecular biology and genomic technologies have allowed us to obtain detailed information on these bacteria. The importance of RSS in environmental technologies requires further consideration.

## 1. Introduction

Hydrogen sulfide from violent movements of the Earth’s crust (e.g., volcanic eruptions) provided the energy, reducing power, and material basis for the origin of life about 3.8 billion years ago [1,2]. The ensuing anoxygenic and oxygenic photosynthesis led the Earth into an era known as the “great oxidation event” (GOE) [3]. The hypothesis of “ox-tox” is that early living organisms evolved antioxidant defense systems (e.g., superoxide dismutase, catalase, peroxiredoxins, thioredoxin, and glutaredoxin) to counteract the abundance of oxygen [4]. Sulfide-based biochemical reactions persist in modern times, not as a primary source of energy, but as a regulator of metabolism and signaling. This basis to create, regulate, and maintain life activities is redox reactions, such as photosynthesis and respiration [2].

**Figure 1:**
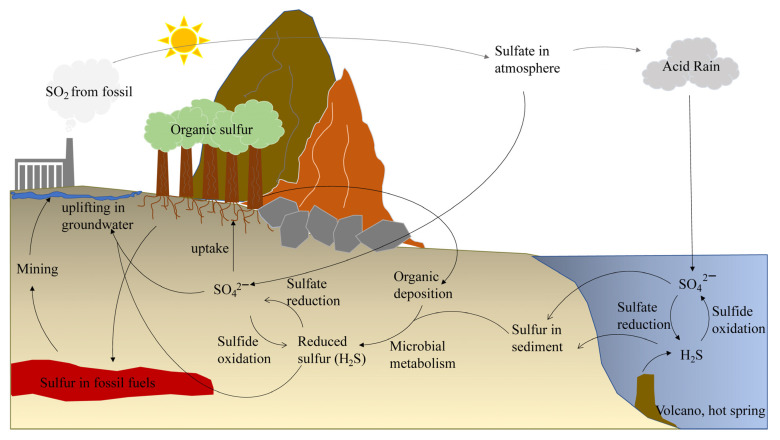
Global sulfur cycle.

Reactive oxygen species (ROS), reactive nitrogen species (RNS), and reactive nitrogen oxides (RNOS) are highly oxidizing, destroying redox-sensitive proteins and enzymes and attacking membranes and DNA [5]. Anti-oxidation is a core topic in physiological and biochemical research, and the attention of researchers has shifted from oxygen to sulfur. Sulfur-containing materials are generally considered to exist naturally as antioxidants (e.g., hydrogen sulfide and glutathione). The definition of reactive sulfur species (RSS) emerged in 2001 and research has focused on physiological, biochemical, and protein molecular functions. Previous reviews described the active chemical properties and physiological effects of RSS [6,7]. The sulfur cycle is an important part of global geochemical cycles (Figure 1) [8,9], but what role does RSS play in the field of environmental technology?

In this review, we detail the relationship between RSS and pollutants, environmental technologies, and metabolic mechanisms. The review emphasizes the prevalence and importance of RSS in environmental technologies and provides an outlook on application prospects and future development of RSS.

## 2. RSS Definition and Relationship with Environmental Management

### 2.1. Origins and Definition of RSS

Giles et al. first defined RSS by presenting it as an oxidative-stress product and juxtaposing it with ROS, RNS, and RNOS [10]. Later, Brannan and Gruhlke amended the definition of RSS to “sulfur-containing molecules capable of oxidizing or reducing the oxidative reactive activity of biological macromolecules under physiological conditions” [11,12]. This definition does not include environmental microorganisms. This is because, for many simple chemoautotrophic microorganisms, the source of energy for survival is inorganic matter or sunlight. In these cases, in addition to hydrogen sulfide, inorganic reduced sulfur substances, such as elemental sulfur (S^0^), can serve as an electron donor or electron acceptor for growth. From this perspective, S^0^ has properties similar to those of RSS, which is susceptible to oxidation or reduction by biological processes. We suggest that RSS should be defined more broadly to include sulfur-containing molecules which are bioavailable and susceptible to redox reactions. The chemical valence states of the sulfur atoms in the typical sulfur-containing compounds are summarized in Table 1. This review is particularly focused on sulfide, S^0^, polysulfide, sulfur dioxide, and sulfate.

### 2.2. Relationship between RSS and Environmental Pollutant Management

Sulfur is the 10th most common element in the universe, the 15th most common element in the Earth’s crust, and the 7th most common element in biology [13]. Its main forms are pyrite (FeS_2_) and gypsum (CaSO_4_) in the ground and free sulfate in the ocean. It has a valence state of −2 to +6, and is more stable at even numbers. H_2_S (−2), the most reduced form of sulfur, is characterized by a rotten-egg odor, typical of RSS. In aqueous environments, it exhibits properties of a dibasic weak acid. Despite the similarity to H_2_O, the transmembrane behavior is different, with H_2_S in the ionic form of HS^−^ by simple free diffusion [14,15]. The anaerobic environment is favorable for the generation and aggregation of H_2_S. With anthropogenic intervention, sulfide-containing wastewater is often observed in industrial plant wastewater, e.g., petrochemical plants, tanneries, synthetic fiber manufacturers, or coal gasification power plants. Therefore, the release of H_2_S into the environment, as dissolved sulfide in wastewater or as H_2_S in flue gases, is controlled for environmental protection.

S^0^ is one of the major sulfur pools in the global sulfur cycle. Chemically generated S^0^ has low water solubility (5 μg/L, 25 °C), with the bio-generated form being hydrophilic and more bioavailable [16]. As a non-corrosive solid that is environmentally friendly and easy to handle and transport, S^0^ is pursued as a target for sulfur-containing pollution treatment. Its commercial value exceeds that of sulfuric acid, even though both can be used in chemical processes and fertilizer production [17].

**Figure 2:**
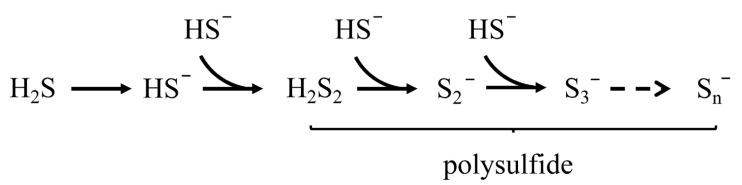
Hypothetical pathway of polysulfide generation.

Polysulfides (RS_n_R, RS_n_H, H_2_S_n_; n ≥ 2), highly reactive chemical intermediates, often accompany the oxidation of sulfides and the bioavailability of sulfur, and are also typical of RSS [18]. The hypothetical polysulfide generation process is shown in Figure 2 [19]. Similar to S^0^, polysulfides can act as both electron acceptors and electron donors. Polysulfides, rather than hydrogen sulfide, play an important role in intracellular antioxidation, persulfide modification, and signaling [20,21,22]. S^0^ and polysulfides are important as intermediate products of the global sulfur cycle in different sulfur reservoirs and isotope fractionations.

SO_2_(+4) is a toxic, colorless, environmental pollutant. It has a wide range of sources, such as coal-fired processes in power plants, incinerators, and boilers. The dispersion of sulfur dioxide gas into the atmosphere causes photochemical smog, acid rain, stratospheric ozone depletion, and fine particulate matter, causing serious harm to ecosystems and corroding the metal components of industrial equipment [23]. Efforts have focused on the development of qualified technologies to eliminate SO_2_ from coal-combustion flue gas. Flue-gas exhaust contains some CO, nitrogen oxides (NO_X_), and small amounts of O_2_ in addition to SO_2_ [24]. Developing technologies for integrated methods of treating multiple greenhouse gases remains a global priority.

Sulfate (+6), one of the main forms of sulfur in nature, is a type of secondary pollutant due to its anaerobic reduction products [25]. Sulfate-laden wastewater is characterized by a long latent period and is difficult to treat. Wastewater with high untreated sulfate levels causes acidification of surface and groundwater, damage to soil structure, and reduction of crop yield [26]. High sulfate concentrations lead to off-flavors (>400 mg L^−1^) and diarrhea (1000–1200 mg L^−1^) [27]. The development of high-sulfate wastewater treatment technologies with solid elemental sulfur as a recovery target is important.

## 3. RSS-Related Bioprocesses for the Treatment of Environmental Pollutants

**Figure 3:**
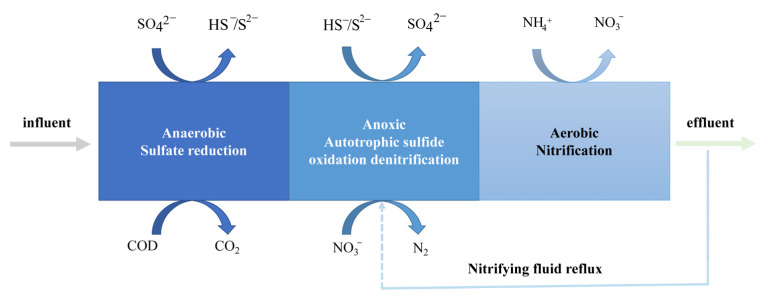
Diagram of SANI process.

A central issue in wastewater treatment is nitrogen removal [28]. Excess nitrogen causes eutrophication, has a toxic effect on aquatic plants and animals, and contaminates drinking water sources [29]. Biological denitrification stands out for its low operating costs and environmental friendliness. In this process, both autotrophic and heterotrophic bacteria play roles separately or together. The SANI (sulfate reduction−autotrophic denitrification−nitrification integrated) process is successful in practical municipal wastewater treatment, especially in terms of energy and sludge reduction (Figure 3) [30]. We review wastewater treatment mediated by sulfur-containing substances, categorized by main sulfur species, focusing on the main functional microorganisms, functional genes, and metabolic mechanisms. In addition to the SANI process, other types of reactors, such as membrane reactors and elemental sulfur packed-bioreactor, are covered. Some of the treatment processes that involve exhaust gas treatment are also discussed.

### 3.1. Sulfur-Reduction-Based Biological Treatment

#### 3.1.1. Sulfate-Reduction Bioreactors

Sulfate can be used as an alternative terminal electron acceptor under anaerobic conditions, except for oxygen, nitrate, Mn (IV), and Fe (III), which provide higher energy yields. Therefore, sulfate reduction is not only an important part of the global sulfur cycle but is also applied in wastewater treatment. The use of sulfate-reducing bacteria (SRB) for the treatment of high-sulfate wastewater is appropriate, given the potential threat of excessive sulfate emissions to the environment. To the best of our knowledge, the sulfate respiration of SRB relies on a variety of electron donors, such as formate, acetate, butyrate, and H_2_, with sulfide as the end product [31,32]. Sulfate-reduction bioreactors are used in single or multi-stage systems for full-depth treatment of wastewater, depending on the purpose of the treatment.

Bio-sulfate-reduction technology for the removal and recovery of valuable metals is critical [33]. Metallic wastewater from acid mine drainage (AMD) and heavy industries, such as metallurgy and steel manufacturing, has low pH and COD (chemical oxygen demand), high sulfate, and high heavy metals [34]. The advantages of using SRB to precipitate metals include (1) SRB have a broad spectrum of pH adaptability and can perform sulfate reduction at low pH to produce sulfides, (2) high sensitivity of sulfide precipitation reactions and high recoverability, and (3) low cost. Treatment of antimony (Sb) mine drainage is regarded as a priority by regulators, and sulfate-reduction bioreactors have great potential for Sb removal [35]. Up to 98.3% antimony removal is achieved in SRB reactors with Fe(II) participation, and soluble Sb(V) is reduced to Sb(III) and precipitated as pyroxene (Sb_2_S_3_) [36]; a typical strain of SRB enriched therein is *Desulfovibrio* sp. Macroscopically, SRB utilizes the organic compounds in wastewater to provide electrons for sulfate reduction, which results in the production of sulfides that combine with metal ions to form insoluble precipitates. These reactions can be expressed by two equations:

2CH_2_O + SO_4_^2−^ + 2H^+^ → H_2_S + 2CO_2_ + 2H_2_O (1)

H_2_S + M^2+^ → MS + 2H^+^ (2)

where M is a metal, e.g., Mn, Pb, Cu, Cd, or Ni. Sulfate reduction is an alkalinity-producing process that is advantageous in biologically neutralizing acidic wastewater and for ecological restoration. The major problems associated with the anaerobic treatment of high-sulfate wastewater are related to the production of sulfides. In addition to the precipitation of metals, sulfide can also be used as a feedstock for subsequent bioreactors. Sulfide oxidation used for wastewater treatment is summarized in Section 3.2.

Besides AMD wastewater, sulfate-reduction biotechnology is applied to other types of wastewater, such as antibiotic-containing pharmaceutical or phenol-containing paper-mill wastewater. Ciprofloxacin (CIP) is a fluoroquinolone antibiotic that is widely used in human and animal manufacturing. It has strong antibacterial effects in the treatment of human tuberculosis and urinary tract and respiratory tract infections, as well as in animal husbandry and farming [37]. Jia et al. found that sulfate-reduction biotechnology has great potential to treat wastewater containing CIP [38]. At low concentrations CIP is adsorbed by secreting extracellular polymeric substances (EPS), thus avoiding the toxic effects of antibiotics on microorganisms—with increasing CIP concentrations, CIP-resistant *Desulfobacter* are enriched. The CIP biodegradation pathway dependent on cytochrome P450 enzymes and acetylases was validated in an SRUSB (sulfate-reducing up-flow sludge bed) reactor [39].

Other examples are phenols and their derivatives present in wastewater from textile, paper, plastic, and cosmetic industries, as well as in industrial phenol leaks and exhaust gases from construction and renovation [40]. Because of their toxicity and carcinogenicity, phenol substances may cause pollution, which has attracted widespread attention from the scientific community and the public. Anaerobic treatment of phenol-containing wastewater is mostly performed in UASB (up-flow anaerobic sludge blanket) reactors [41]. Guo et al. achieved up to 90% phenol removal using a UASB reactor based on sulfate reduction [42]. Sequencing 16s DNA showed that *Clostridium* spp. and *Desulfotomaculum* spp. were the major phenol-degrading bacteria. Dephosphorylation and acidification are known to be the main pathways of phenol biodegradation [43].

#### 3.1.2. Sulfate-Reducing Bacteria (SRB) and Molecular Mechanisms

Sulfate-reducing bacteria, an artificial taxonomic designation according to function, comprise a diverse group of anaerobic microorganisms with a wide range of fermentation-product metabolism capabilities [44,45]. SRB are distributed in more than 220 species in 60 genera of five phyla of bacteria and two divisions of archaea [46,47]. Bacteria taxa include *Desulfovibrio, Desulfotomaculum,* and *Desulfosporomus* in phylum Firmicutes, *Thermodesulfovibrio* of phylum Nitrospira, and *Thermodesulfobacterium.* For archaea, the euryarchaeota genus *Archaeoglobus* and the two crenarchaeotal genera *Thermocladium* and *Caldivirga* are dominant. The dominant SRB vary in different bioreactors. For example, in an expanded granular sludge bed (EGSB) reactor capable of carbon, nitrogen, and sulfur co-removal operated by Chen, the dominant strain of SRB was *Desulfomicrobium* sp. [48]. *Desulfomicrobiaceae* and *Desulfobulbaceae* are the two dominant SRB taxa in sulfate-reduction and organic-matter-removal units [49]. Two new species were defined in the sulfate-reducing ammonia anaerobic oxidation (SRAO) process, *Anammoxoglobus sulfate* and *Bacillus benzoevorans*, which possess the ability to simultaneously eliminate ammonia and sulfate [50].

**Figure 4:**
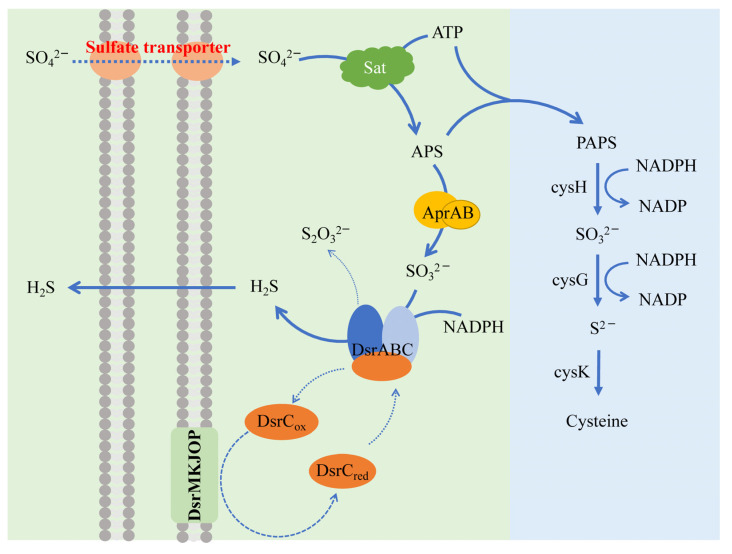
Two sulfate-reduction pathways. The left is the dissimilatory sulfate-reduction pathway, where the product is hydrogen sulfide, and the right is the assimilatory sulfate reduction, where sulfide is utilized for cysteine synthesis.

Regardless of the environment or bioreactor, a common set of dissimilatory sulfate-reduction pathways (also called ‘‘sulfate respiration’’) are shared by functional SRB as shown in Figure 4 [51,52,53]. Sulfate is taken up from the environment via sulfate transporters and activated by the enzyme ATP sulfurylase (Sat) to form adenosine-5′-phosphosulfate (APS). Then APS is reduced to sulfite through adenylyl-sulfate reductase (Apr), which accepts electrons from the electron transport complex (ETC) in the membrane. The dissimilatory (bi)sulfite reductase (DSR) complex further reduces the (bi)sulfite to H_2_S, which diffuses passively out of cell membranes. Besides the dissimilatory sulfate-reduction pathway, there is an assimilatory sulfate pathway in SRB [54,55]. Both share the same initial step of sulfate activation by ATP—the difference is that assimilatory sulfate reduction requires the transfer of phosphate to adenosine-5′-phosphate sulfate (APS) by adenylate kinase to produce phosphoryl adenosine-5′-phosphate sulfate (PAPS). This continues to be decomposed by NADPH_2_ to produce SO_3_^2−^ and, finally, a cysteine is formed from SO_3_^2−^ by sulfite reductase.

The enzymatic reaction of sulfate reduction is reversible due to the intermediate products and substrate concentrations. This explains sulfur isotope fractionation [56,57]. Genes dsr*A* and dsr*B* are regarded as the characteristic key functional genes of SRB, and they have been used to investigate the distribution and abundance of SRB in colonies [58]. Specific inhibition of sulfate reduction by molybdate or selenate has been experimentally demonstrated, and this has been used to study the contribution of different electron donors to sulfate reduction [59].

In addition to temperature and pH, two basic physicochemical indicators that directly affect the activity of SRB substrate carbon supply are to be considered for sulfate-reduction biotechnology: (1) SRB are mostly heterotrophic in metabolism and (2) different types of SRB utilize different carbon sources [44]. Therefore, the provision of suitable carbon sources is of significance to improve the efficiency of SRB reactors. Scientific research mostly uses a single carbon-source culture, but it is expensive. In large-scale applications, such as industrial wastewater treatment, alternative efficient and inexpensive carbon source supplies must be considered. Mixing multiple carbon sources is common. Steel slag, sugarcane bagasse, fruit and vegetable wastewater, and sugar by-products have been introduced as cheap carbon sources [60,61]. In anaerobic wastewater treatment, methanogenic bacteria compete with sulfate-reducing bacteria for hydrogen and acetic acid (both are prerequisites for methane formation and electron donors for sulfate reduction) [62,63]. Providing suitable reaction conditions and controlling the activity of methanogenic bacteria are also important to improve sulfate-reducing bioreactors [64].

#### 3.1.3. S^0^-Based Reduction Bioreactors

Sulfur-packed bioreactors have significant advantages in treating both high-rate COD wastewater and low C/N ratio domestic wastewater by avoiding high activated-sludge yields [65,66]. Sulfur-packed bioreactors can be categorized into two major types according to the electron valence change of sulfur. One is as electron acceptors, mainly used in the treatment of high-organic-carbon wastewater and hazardous metal-laden wastewater [65,67]. The other is as electron donors for in-depth denitrification of drinking-water resources and wastewater with a low C/N ratio (see Section 3.2.3) [68]. These technologies provide a more cost-effective solution to the environmental problems in current wastewater treatment.

The S^0^-based reduction bioreactor is an efficient anaerobic wastewater treatment process that reduces sludge production and avoids the excess activated sludge problem commonly faced by wastewater plants [69]. A laboratory-scale sulfur-reducing anaerobic fluidized bed (SRAFB) reactor built by Zhang et al. achieved high organic removal rates with a sludge yield of only 16% (VSS per kg COD) [70]. Sulfide in the effluent can be recovered by micro-aeration biological treatment, an internal sulfur cycling process (ISC). An ISC system achieved 94% removal at 300 mg/L COD after 200 days of continuous operation, and 76% recovery of sulfide in the effluent was recovered in the form of elemental sulfur after 200 days of continuous operation [71].

Emerging sulfur-reduction biotechnology requires only two electrons for the sulfidation of elemental sulfur, theoretically reducing organic consumption by 75%. Sulfur reduction can reduce organic carbon by 66–80% compared to sulfate reduction when producing equivalent amounts of sulfide [67]. Li et al. performed a pilot-scale sulfur reduction bioreactor to handle practical domestic wastewater, coupling Cu-laden electroplating wastewater treatment [72]. The results achieved 99% removal of Cu^2+^, indicating that sulfur reduction is a sustainable sulfide generation technology with great potential for application.

Mercury and arsenate removal is also critical for S^0^-based reduction bioreactors. Arsenite (III) is more mobile and toxic than arsenate (V) and both are culprits of arsenic contamination in groundwater. Sulfide precipitation is the ideal means of biological arsenic removal [73]. Because sulfate reduction is alkali-producing, the by-product thioarsenite (As(OH)S_2_^2−^) is produced [74]. Therefore, sulfur-reduction technology under acidic conditions is considered a prospective alternative because it produces large amounts of sulfide while minimizing pH increases. Sun et al. verified that an S^0^-based reduction bioreactor could produce high sulfide yields (0.42 ± 0.2 kg S/m^3^-d) under acidic conditions (pH~4.3) while achieving 99% removal of arsenite without the formation of soluble thioarsenite [75].

2H_3_AsO_3_ + 3HS^−^ → As_2_S_3_ + 3H_2_O + 3OH^−^ (3)

H_3_AsO3 + HS^−^ + 2H^+^ → AsS + 3H_2_O (4)

As_2_S_3_ + HS^−^ + 3OH^−^ → 2As(OH)S_2_^2−^ + H_2_O (5)

Sulfur-reduction technology also has the potential for treating mercury (II) in aqueous environments. Mercury (II) is highly toxic and can be removed by the formation of insoluble precipitates with biogenic sulfides. Sulfate reduction, however, does not achieve desired mercury removal because SRB promotes the production of the more toxic methylmercury (MeHg) in the presence of organic matter and sulfate [76]. Wang et al. performed successive experiments on mercury-laden wastewater and found that the use of S^0^-based reduction bioreactors completely removed mercury (II) (up to 50 mg/L) without forming neurotoxic MeHg [77]. However, the causes and mechanisms for no by-product MeHg production in this process are not clear.

Sulfur-packed bioreactors have also been used in flue-gas treatment. SO_2_ has high solubility (11.29 g SO_2_/100 g H_2_O), whereas NO, which is the major component of NO_x_, does not (0.00618 g NO/100 g H_2_O). The traditional physical−chemical desulfurization and denitrification approach is wet flue-gas desulfurization (WFGD) for SO_2_ removal with selective catalytic reduction (SCR) of nitrogen oxides [23]. Reducing substances produced during wastewater treatment, such as ammonia, nitric oxide, and hydrogen sulfide, have been shown to act as reducing agents for flue-gas desulfurization and denitrogenation. Sun et al. developed a simultaneous catalytic desulfurization and denitrogenation (SCDD) technology based on sulfur cycling [78]. This technology takes the organic matter in wastewater as an electron donor and obtains high-rate sulfide by biological sulfur reduction; the resulting low-cost reductant (hydrogen sulfide) removes 90% of SO_2_ and NO from the flue gas, and the end product was elemental sulfur that was non-toxic and had economic recovery value.

Polysulfides have been found to participate in and accelerate the sulfur reduction in S^0^-based reduction bioreactors. As a product of the nucleophilic attack of sulfur hydrogen ions on elemental sulfur, polysulfides are a key intermediate in sulfur reduction and they enhance the bioavailability of sulfur. Polysulfides were also found by Zhang et al. in their laboratory-scale, sulfur-reducing anaerobic fluidized bed (SRAFB) bioreactor for wastewater treatment [70]. The small initial amount of sulfide promoted the production of polysulfide, which accelerated the reduction of elemental sulfur, forming a polysulfide-mediated self-accelerating chain reaction. Qiu et al. suggested that a novel polysulfide-involved SADN (PiSADN) process achieved a high rate of autotrophic nitrate removal [79]. In this process, sulfur disproportionation is considered to be the key to driving PiSADN, where disproportionation generates sulfides, which, in turn, promote the formation of polysulfides.

HS^−^ + (n − 1) S^0^ → S_n_^2−^ + H^+^ (6)

4S^0^ + 4H_2_O → SO_4_^2−^ + 3HS^−^ + 5H^+^ (7)

Δ*G*^0^ = 240.2 kJ/mol

#### 3.1.4. Sulfur-Reducing Bacteria (S^0^RB) and Molecular Mechanisms

Elemental sulfur reduction to sulfide coupled with inorganic phosphorylation of ADP is known as sulfur respiration [80]. Since the discovery of sulfur respiration in *Desulfuromonas acetoxidans*, more bacteria that can catalyze elemental sulfur reduction have been discovered. Sulfur-reducing bacteria (S^0^RB) are distributed in both archaea and bacteria and have a wide range of habitats in nature, from extremely acidic hot seawater to superheated seafloor vents [80,81]. Because of this, the metabolism of S^0^RB exhibits high variability (Table 2).

**Figure 5:**
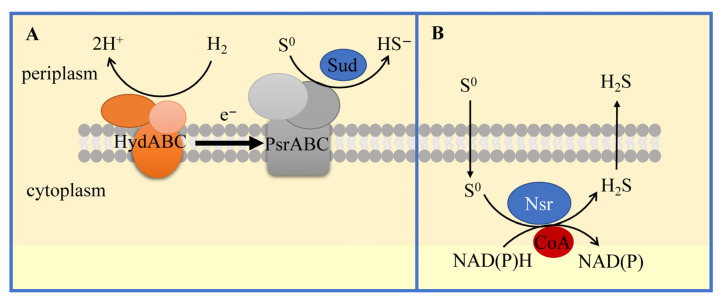
Two known mechanisms of sulfur respiration. (**A**) The Psr pathway where electrons for sulfur reduction are derived from hydrogenase. (**B**) The Nsr pathway where electrons for sulfur reduction come directly from NAD(P)H and require the participation of coenzyme A.

There are at least two known mechanisms of sulfur respiration in S^0^RB (Figure 5) [96]. One is found in *Wolinella succinogenes*, in which the [NiFe]-hydrogenase (HydABC) oxidizes H_2_ and transfers electrons via methyl quinone to the periplasmic membrane-bound polysulfide reductase, PsrABC [80]. PsrA is responsible for polysulfide reduction to H_2_S, PsrB is an [FeS] electron transfer protein, and PsrC is a quinone-containing membrane anchor. In addition, a polysulfide transferase (Sud) protein is thought to be involved in the acquisition of sulfides from protons and sulfur. The other is the NAD(P)H elemental sulfur reductase (Nsr) that uses elemental sulfur as a substrate directly, rather than polysulfides, to reduce elemental sulfur by oxidizing NAD(P)H and releasing H_2_S [96].

### 3.2. Sulfur-Oxidation-Based Biological Treatment

#### 3.2.1. Sulfide-Oxidation Bioreactors

Sulfide is highly reductive and serves as an energy source for some chemoautotrophic microorganisms. It is found in many scenarios, such as anaerobic treatment effluent of sulfate-laden wastewater, sulfidogenic treatment of acid mine drainage, petroleum refining industries, and pharmaceutical wastewater [34,37]. In the geochemical cycle, sulfide is re-oxidized back to sulfate via various oxidants, such as oxygen, nitrate, Mn (IV), Fe (III), and other chemical oxidants or bio-oxidizers, such as reductive sulfur substances oxidizing bacteria (SOB), through different sulfur intermediates (polysulfide, elemental sulfur, sulfite, thiosulfate, etc.). The degree of sulfide oxidation depends on the number of available chemical oxidants (e.g., oxygen and nitrate) and the species of SOB [62]. SOB is a group of microorganisms that utilize reduced sulfur substances (sulfide, elemental sulfur, or thiosulfate) and whose oxidation products are higher-valence sulfur-containing substances or sulfates. Due to the diversity of their nutrient metabolism types, they have long been used in wastewater treatment [97]. Practical wastewater systems contain organic carbon, nitrate nitrogen, and ammonia nitrogen in different concentrations, besides sulfurous substances. Therefore, various simultaneous desulfurization and denitrification technologies have been developed to deal with sulfurous wastewater pollution [98].

Denitrifying functional microorganisms are classified into two main groups depending on the electron donor. Processes using organic material are called heterotrophic denitrification (HD) and those using inorganic materials (e.g., Fe^2+^, Mn^2+^, H_2_, S^2−^, and S^0^) are called autotrophic denitrification (AD). The former has the advantage of rapid denitrification but disadvantages include sludge production, N_2_O emissions, and exogenous supplemental carbon sources. AD decreases sludge yield but has a long start-up period and slow bacterial growth [99]. The choice of autotrophic or heterotrophic denitrification, or a combination, depends on the type of wastewater being treated.

Autotrophic denitrification technology with sulfide as an alternative electron donor is applied to the desulfurization of biogas and denitrification of low C/N ratio wastewater. This can avoid the exogenous addition of carbon sources, and the intermediate oxidation product (elemental sulfur) is not a secondary pollutant and has economic value [100]. Therefore, the final treatment of sulfur-containing wastewater is often targeted at elemental sulfur. As shown in Figure 3, AD is the core technology unit in the SANI system, in which sulfide and nitrate are synchronously converted by microorganisms into sulfate and N_2_, thus achieving the goal of harmless and resourceful wastewater.

Biogas, a biomass energy source, has many advantages, such as high combustion value, simple preparation, sufficient raw materials, and low pollution; however, the formation of hydrogen sulfide as a by-product is inevitable [101]. Although the concentration of H_2_S is low, it will have a strong corrosive effect on metal pipes, instruments, internal combustion engines, etc. Moreover, it will produce SO_2_ after combustion, which will cause pollution. Therefore, desulfurization is an essential part of biogas purification [102,103]. The coupling of biogas desulfurization with deep denitrification of wastewater is increasingly common.

Similar to sulfur-containing wastewater treatment, biodesulfurization uses SOB to convert H_2_S in biogas into elemental sulfur or sulfate. Wang et al. proposed a new process using autotrophic denitrification coupled with biogas desulfurization [104]. The process uses H_2_S in biogas as the electron donor for wastewater denitrification and achieves deep nitrogen removal from wastewater and simultaneous purification of biogas without an additional carbon source. Even if the N/S parameters change, the removal rate of elemental nitrogen in the effluent can reach 100% and the removal rate of hydrogen sulfide remains above 91%.

The combination of autotrophs and heterotrophs has significant advantages in wastewater treatment, such as increasing the stability of the reactor network, compensating for insufficient organic carbons, and minimizing sludge yields. On this basis, integrated autotrophic heterotrophic denitrification (IAHD) is proposed for the treatment of organic wastewater containing nitrogen and sulfide, i.e., simultaneous carbon, nitrogen, and sulfur removal. Reyes-Avila et al. achieved simultaneous removal of nitrate (to N_2_), sulfide (to S^0^), and carbon (acetate to CO_2_) in a continuously stirred tank reactor (CSTR) using an incubated autotrophic heterotrophic symbiotic system [105]. The maximum removal rates were 0.209 kg N m^−3^ d^−1^, 0.294 kg S m^−3^ d^−1^, and 0.303 kg C m^−3^ d^−1^. Chen et al. used an EGSB to achieve high rates of bioconversion in synthetic wastewater, at loading rates of 3.0 kg S m^−3^ d^−1^, 1.45 kg N m^−3^ d^−1^, and 2.77 kg Ac m^−1^ d^−1^ [106]. Zhang et al. investigated the contribution of autotrophic and heterotrophic bacteria in an IAHD system and found that *Thiobacillus* was the key autotrophic desulfurization and denitrification bacterium at low sulfide levels, while other heterotrophic bacteria, such as *Azoarcus* and *Pseudomonas*, functioned at high sulfide concentrations [107].

Huang et al. achieved 78% recovery using a UASB reactor while ensuring 100% carbon, nitrogen, and sulfur co-removal [108]. Further, they developed a compact, biofilm-forming, membrane-filtration reactor (BfMFR) aimed at the rapid separation of the generated elemental sulfur from the biofilm by membrane filtration [109]. The high sulfur generation efficiency (98% on average) was stably maintained with feed water concentrations of 115, 120, and 100 mg/L for acetic acid, nitrate, and sulfide. Researchers found that the genera *Thauera*, *Arcobacter*, *Pseudomonas*, *Azoarcus*, *Ochrobactrum*, *Alkiflexus*, and *Thiobacillus* were prevalent and they were the core genera of denitrification desulfurization system [109].

#### 3.2.2. Sulfide-Oxidation Bacteria (SOB) and Molecular Mechanisms

The biological oxidation of sulfides is an ancient metabolic mode and a common chemical reaction in extreme environments such as volcanoes and hot springs. The microorganisms that dominate these oxidation reactions are diverse and include various trophic groups of bacteria and archaea. Table 3 summarizes the taxonomy, nutrient types, and enzymes of several representative SOBs [110,111,112,113,114].

**Figure 6:**
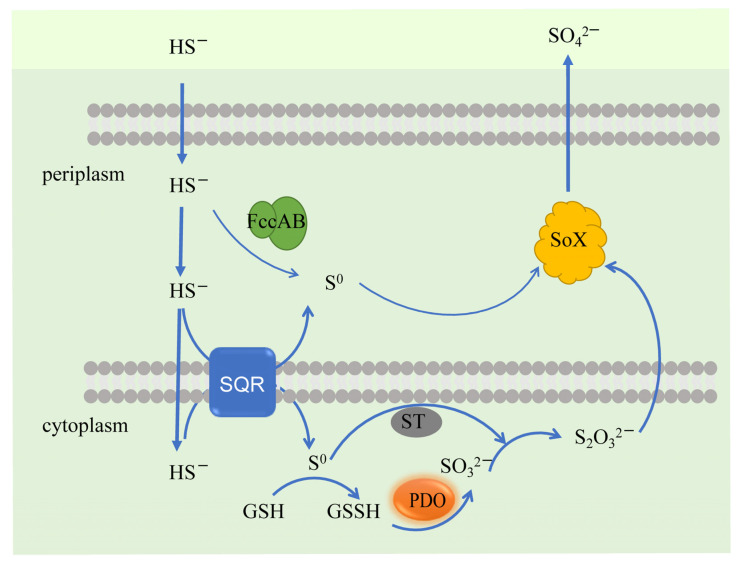
Biological oxidation pathway of sulfides. FCSD and SOX are located in periplasmic space and the SQR/PDO/ST pathway is located in the cytoplasm.

Colorless sulfur bacteria include *Paracoccus*, *Hyphomicrrobium*, *Alcaligenes*, *Pseudomonas*, *Ochrobactrum,* and *Hydrogenobacter*. *Thiobacillus denitrificans* is the most well-studied chemoautotrophic sulfide-oxidizing bacterium, capable of sulfide oxidation under aerobic and anaerobic conditions [127]. Primary sulfide-oxidation pathways include the sulfide−quinone oxidoreductase (SQR/PDO/ST) system, flavin cytochrome c dehydrogenase (FCSD), and Sox multi-enzyme oxidation system. Among them, SQR and FCSD are the dominant types of sulfide oxidases (Figure 6). There are six SQR systems distributed in animals, plants, and microorganisms [111,128]. SQR relies on its cofactor FAD to oxidize sulfide to zero-valent sulfur, and the resulting electrons enter the respiratory chain via coenzyme Q or methyl naphthoquinone on cell membranes. The resulting zero-valent sulfur reacts spontaneously with GSH in the presence of a suitable receptor (e.g., GSH) to form glutathione persulfide (GSSH), which is then oxidized to sulfite by persulfide dioxygenase (PDO) [129]. The zero-valent sulfur is temporarily bound to the conserved cysteine of SQR in the absence of a suitable receptor, and as the sulfide is oxidized; the zero-valent sulfur bound to SQR is eventually shed as S_8_. By contrast, FCSD is a heterologous flavoprotein dimer formed by the binding of two c-type cytochrome subunits encoded by the *fccA* and *fccB* genes, which are generally found in the microbial periplasmic space [130]. FCSD differs from the electron acceptor of SQR in that it uses cytochrome c as an electron acceptor to oxidize sulfide to zero-valent sulfur. The FCSD system is thought to be useful in areas of low sulfide concentration and, therefore, SQR is generally considered to be the primary sulfide-oxidation system (especially in high sulfide environments) [131]. Therefore, *sqr*, *fccA*, *fccB*, *pdo*, and *sox* are often queried as key characteristic genes or proteins in the distribution and diversity analysis of SOB.

The optimization of reactor operating parameters, such as temperature, pH, HRT, N/S ratio, and C/N ratio of the influent, directly affects the operating effectiveness of denitrification sulfide removal [132]. The rate of microbial-catalyzed sulfide oxidation is several orders of magnitude higher than chemical oxidation [133,134]. Researchers have demonstrated that microaerobic conditions (DO in the range of 0.2–1 mg L^−1^) can improve the sulfide tolerance of functional bacteria, promote the efficiency of biodesulfurization, and increase the elemental sulfur yield [135,136,137]. Macrogenomic results show that micro-oxygen promoted the abundance of genes responsible for sulfide metabolism (*sqr*, *glpE* (a typical sulfotransferase gene in *Escherichia coli*), *pdo*, *sox,* and *cysK* (Figure 4)) [138]. The formation of polysulfides is inevitable during the oxidation of sulfides [139].

#### 3.2.3. S^0^-Based Oxidation Bioreactors

S^0^-based oxidation bioreactors are primarily applied for the intensive denitrification of low C/N ratio wastewater or groundwater for economic reasons. More importantly, sulfur autotrophic denitrification (SADN) emits less N_2_O than heterotrophic denitrification [140]. Sahinkaya performed a new SADN using a membrane bioreactor (MBR) to remove nitrate from drinking water [141]. Complete denitrification was achieved when the influent nitrate concentration was 25–50 mg NO^3^-N/L and the HRT was as low as 5 h. Zhang et al. achieved a removal efficiency of 4.0 g NO_3_-N/L·d using a novel sulfur-oxidizing autotrophic denitrifying anaerobic fluidized bed membrane bioreactor (AnFB-MBR). They found *Thiobacillus*, *Sulfurimonas,* and *Ignavibacteriales* to be the dominant sulfur-oxidizing bacterial genera [66]. Denitrification is alkalinity-depleting, so cheap and easily available materials, such as CaCO_3_ or crushed oyster shells, are good choices to neutralize alkalinity. SADN has been applied in wastewater treatment plants and for the production of drinking water [142].

S^0^-based oxidation bioreactors have also been applied for chromium removal from drinking water. Chromium contamination is not uncommon in industrial wastewater and groundwater [143]. In nature, hexavalent (VI) and trivalent (III) chromium are the main forms, the former is water soluble and strongly carcinogenic, and the latter is insoluble in neutral conditions. The main method of chromium removal from water bodies is to reduce Cr(VI) to Cr(III) [144]. After 60 days of operation, 92.9% removal of chromate was achieved with the reactor using elemental sulfur as the only electron donor.

#### 3.2.4. Sulfur-Oxidation Bacteria (S^0^OB) and Molecular Mechanisms

**Figure 7:**
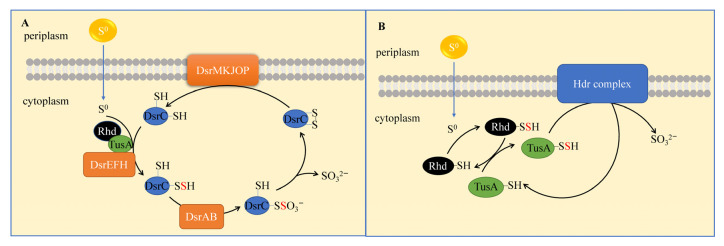
Biological oxidation pathway of elemental sulfur. (**A**) The rDSR pathway and (**B**) the Hdr pathway.

Sulfur-oxidizing bacteria (S^0^OB) are microorganisms capable of directly using elemental sulfur as an electron donor. Due to the relevance of the metabolism of reduced sulfur species (sulfide, sulfite, thiosulfate), S^0^OB can oxidize the above-mentioned reducing sulfur species. Here, we review two known metabolic pathways for microbial elemental sulfur oxidation: the rDSR (reverse dissimilatory sulfite reductase) pathway and the Hdr (heterodisulfide reductase) pathway (Figure 7). The rDSR pathway involves several enzymes in dissimilatory sulfate reduction as mentioned in previous sections [145]. The sulfur atoms in elemental sulfur being sequentially transferred to the active site of rDSR through proteins Rhd, TusA, DsrEFH, and DsrC. The two active Cys of protein DsrC and the received sulfur atoms form a trisulfide peroxide catalyzed by the membrane-bound protein complex DsrMKJOP, and SO_3_^2−^ is produced by the DsrAB protein. In this process, low-molecular-weight organic persulfides (e.g., glutamine persulfide) are carriers for the transfer of sulfur from the periplasmic space to the cytoplasm. The Hdr pathway is a sulfur-atom-transfer pathway, similar to the rDSR pathway, which produces sulfite [146]. The difference is that the Hdr complex is a membrane-bound protein containing at least five subunits.

## 4. Prospects and Conclusions

This study highlights the scientific and environmental aspects of relying on the sulfur cycle for pollutant treatment by reviewing current advanced biotechnologies and the available molecular biological knowledge. Yet, there remains a gulf between currently known molecular mechanisms and practical biotechnological guidance. A better interplay between the two should be addressed in the future for both basic theoretical research and practical engineering applications. The relevant biological principles and mechanisms in biological treatment need to be optimized by calibrating operating parameters and elucidating more efficient microbial pathways.

RSS are involved in several biotechnological processes as an important intermediate in the microbially driven sulfur cycle. One of the challenges of RSS is the interconversion of different sulfur species through redox reactions, leading to the inability to accurately quantify them, especially polysulfides. The role played by RSS in environmental technology research is also complicated by the oxidative-stress products of functional microorganisms in bioreactors and their interactions with contaminants.

Some substantial advances have been made in sulfur-cycle-based biotechnology for wastewater treatment. A variety of sulfur-packed bioreactors are emerging and the development of single-stage bioreactors for the simultaneous removal of multiple pollutants is a future research direction. Sulfur-packed reactors show their superiority, but safety during transportation and storage should not be ignored.

## Figures and Tables

**Table 1:** Representative sulfur-containing substances in different valence states.

Component	Chemical Formula	Valence of Sulfur
Inorganic sulfur species:		
Sulfide	H_2_S/HS^−^/S^2−^	−2
Pyretic sulfur	FeS/FeS_2_	−2 and −1
Inorganic polysulfides	H-S_n_-H/S_n_^2−^ (n ≥ 2)	−1 and 0
Elemental sulfur	S/S_8_/S^0^	0
Thiosulfate	S_2_O_3_^2−^	+2
Sulfur dioxide	SO_2_	+4
Sulfite	SO_3_^2−^	+4
Sulfate	SO_4_^2−^	+6
Organic sulfur species:		
Reduced organic sulfur compounds	Cysteine, methionine	−2
Organic polysulfides	R-S_n_-R/R-S_n_H (n ≥ 2)	0

**Table 2:** Representative archaea and bacterial members of sulfur respiration.

Taxonomic Category	Electron Donor	Reference
**Archaea**		
Crenarchaeota:		
*Acidianus*	H_2_	[82]
*Thermoproteus*	H_2_, peptides, maltose, formate, fumarate, ethanol, malate, methanol, glycogen, starch, amylopectin, formamide	[83]
Euryarchaeota:		
*Pyrococcus*	Complex substrates, amino acids, starch, maltose, pyruvate	[84]
*Methanococcus*	H_2_, formate	[85]
**Bacteria**		
*Aquifex*	H_2_, sulfur, thiosulfate	[86]
*Desulfurobacterium*	H_2_	[87]
*Desulfuromonas*	Acetate, pyruvate, ethanol	[88]
*Desulfuromusa*	Acetate, propionate	[89]
*Fervidobacterium*	Sugars, pyruvate, yeast extract	[90]
*Geobacter*	Acetate	[91]
*Sulfospirillum*	H_2_, formate	[92]
*Thermotoga*	Sugars, peptone, yeast extract, bacterial and archaeal cell homogenates	[93]
*Thermosipho*	Yeast extract, brain heart infusion, peptone, tryptone	[94]
*Wolinella*	H_2_, formate	[95]

**Table 3:** Representative strains of sulfide-oxidizing bacteria and their metabolic features.

Taxonomic Category	Representative Species	Metabolic Features	Sulfur Oxidation Genes	Distributed Environment	Reference
GSB Chlorobi	*Chlorobaculum tepidum*, *Chlorobaculum thiosulfatiphilum*	Obligate phototrophy; S^2–^, S^0^, or S_2_O_3_^2−^ as e^−^ donors for reduction of CO_2_; extracellular S^0^ globules; potential mixotrophy	SoxXAYZB, APS reductase, Qmo complex, and Fcc	Anaerobic waters, oceans, soils, the Yellowstone hot springs and sediments	[115,116]
PSB Chromatiaceae	*Allochromatium warmingi* *Isochromatium buderi*	Photoautotrophy except for *Rheinheimera* spp.; S^2−^ and S^0^ as e^−^ donors of photosynthesis; intracellular S^0^ globules	-	Oceans, stagnant aquifers, eutrophic lakes with water bodies, and extreme environments rich in sulfides	[117,118]
Ectothiorhodospiraceae	*Allochromatium vinosum* *Ectothiorhodospira vacuolata*	Oxidation of S^2−^ for all the members; extracellular S^0^ globules; polysulfides under alkaline conditions; some can oxidize S_2_O_3_^2−^ to SO_4_^2−^	SoxXAYZB, Sqr, DsrABEFHCMKLJOPNRS, APS reductase, and Fcc		[119,120]
PNSB Alphaproteobacteria	*Rhodopseudomonas palustris*	The preferred photoheterotrophy under anaerobic conditions; photolithoautotrophy with S^2−^/S_2_O_3_^2−^	SoxXAYZBCD, SoxEF, and Sqr	Waste ponds, coastal lagoons and other aquatic-habitat stagnant areas, sediments, wet soils, and rice paddies	[121]
Betaproteobacteria	*Rhodocyclus purpureus*	Chemoorganotrophy/chemolithoautotrophy under aerobic or microaerobic conditions	-		[122]
CSB Alphaproteobacteria	*Paracoccus* spp.	Facultative chemolithoautotrophy; oxidation of S^2−^, S^0^, S_2_O_3_^2−^, or SO_3_^2−^ to SO_4_^2−^	SoxXAYZBCD and SoxEF	Activated sludge, wastewater treatment systems, farmland, and natural ecological environment such as orchards	[123]
Acidithiobacillia	*Acidithiobacillus ferrooxidans*	Obligate chemolithoautotrophy; oxidation of S^0^, S_2_O_3_^2−^, or S_4_O_6_^2−^ by the incomplete Sox system; S^0^ globules as intermediates	SoxXAYZB and Sqr		[124]
Gammaproteobacteria	*Thiomicrospira crunogena*	Obligate chemolithoautotrophy; extracellular S^0^ globules under low oxygen/pH; transient accumulation of SO_3_^2−^ or polythionate during S^0^ globules or S_2_O_3_^2−^ oxidation	SoxXAYZBCD and Sqr		[125]
Gammaproteobacteria	*Beggiatoa* spp.	Chemolithoheterotrophy/mixotrophy; intracellular S^0^ globules	Dsr, Sqr, and APS reductase		[126]

## Data Availability

No data was used for the research described in the article.
